# The supersaturation perspective on the amyloid hypothesis

**DOI:** 10.1039/d3sc03981a

**Published:** 2023-10-16

**Authors:** Diana Portugal Barron, Zhefeng Guo

**Affiliations:** a Department of Neurology, Brain Research Institute, Mary S. Easton Center for Alzheimer's Research and Care, David Geffen School of Medicine, University of California, Los Angeles Los Angeles CA USA zhefeng@ucla.edu

## Abstract

Development of therapeutic interventions for Alzheimer's over the past three decades has been guided by the amyloid hypothesis, which puts Aβ deposition as the initiating event of a pathogenic cascade leading to dementia. In the current form, the amyloid hypothesis lacks a comprehensive framework that considers the complex nature of Aβ aggregation. The explanation of how Aβ deposition leads to downstream pathology, and how reducing Aβ plaque load *via* anti-amyloid therapy can lead to improvement in cognition remains insufficient. In this perspective we integrate the concept of Aβ supersaturation into the amyloid hypothesis, laying out a framework for the mechanistic understanding and therapeutic intervention of Alzheimer's disease. We discuss the important distinction between *in vitro* and *in vivo* patterns of Aβ aggregation, the impact of different aggregation stages on therapeutic strategies, and how future investigations could integrate this concept in order to produce a more thorough understanding and better treatment for Alzheimer's and other amyloid-related disorders.

## Abstract 2

The current amyloid hypothesis does not capture the full complexity of Aβ aggregation. Here we lay out a supersaturation framework to better understand the molecular mechanism of Alzheimer’s disease and to develop more effective treatment strategies.

## Graphical abstract

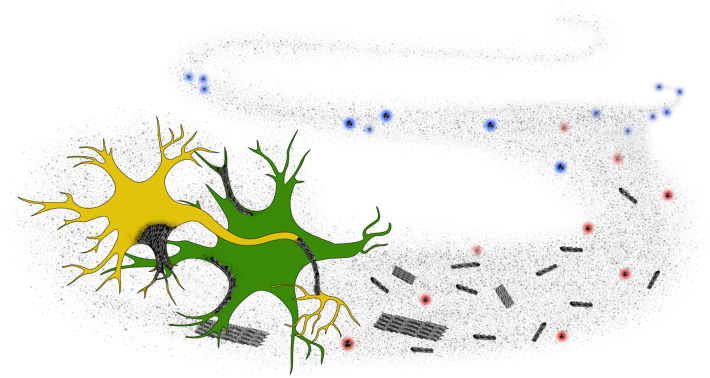

## The amyloid hypothesis

Protein aggregation is associated with a wide range of human disorders such as Alzheimer's disease and type 2 diabetes.^1,2^ The end-product of protein aggregation in these disorders is called amyloid,^3^ and the amyloids formed by different proteins share some common properties such as binding to thioflavin T^4,5^ or cross-β structures.^6,7^ Recent breakthroughs in cryo-EM have led to the elucidation of the structures of many amyloid proteins, showing a diverse structural landscape.^7,8^

Alzheimer's disease has two main pathological hallmarks, the senile plaques consisting of the Aβ protein and the neurofibrillary tangles that are composed of tau.^9–12^ Aβ protein is produced from the sequential cleavage of amyloid precursor protein by β- and γ-secretases.^13^ The γ-secretase cleavage generates two main types of Aβ proteins: the 40-residue Aβ40 and the 42-residue Aβ42, with Aβ42 having two extra amino acids at the C-terminus. Although the overall concentration of Aβ40 is several fold more than that of Aβ42,^14,15^ the main component of the senile plaques is Aβ42.^16,17^

In 1992, Hardy and Higgins^18^ presented the amyloid hypothesis, which states that Aβ, “the main component of the plaques, is the causative agent of Alzheimer's pathology, and that the neurofibrillary tangles, cell loss, vascular damage, and dementia follow as a direct result of this deposition.” Over the years, the amyloid hypothesis has been constantly re-evaluated in light of new experimental discoveries,^19–21^ and has remained as the prevailing theory guiding therapeutic development for Alzheimer's disease.^22,23^ One notable development is the inclusion of Aβ oligomers in the amyloid hypothesis.^24^ Mechanistic understanding of Aβ aggregation in terms of primary and secondary nucleation suggests that amyloid fibrils catalyze the formation of oligomers,^25^ linking oligomers to the overall process of Aβ aggregation.

While earlier failures of anti-Aβ clinical trials have led to criticism of the amyloid hypothesis, the full FDA approval of anti-Aβ antibody lecanemab (marketed as Leqembi) in July 2023 was a turning point in Alzheimer's research.^26^ Unlike the controversial aducanumab (Aduhelm),^27,28^ the findings of lecanemab are straightforward and robust. In the phase 3 trial, lecanemab slowed cognitive decline by 27% on the primary endpoint and also met all key secondary endpoints.^29^ The data from the lecanemab trial are widely considered as a validation of targeting Aβ aggregates as a disease-modifying therapy.^30,31^ The phase 3 trial data of donanemab, an antibody targeting pyroglutamated Aβ, show that donanemab treatment slowed clinical decline by 35% and met all secondary endpoints, further demonstrating the clinical benefits of anti-amyloid therapy.^32^

## Basic concepts of supersaturation in the context of Aβ aggregation

Supersaturation is a well-known concept in the field of protein crystallization, which, like protein aggregation,^33^ is a nucleation-dependent polymerization process.^34,35^ Supersaturation is a non-equilibrium state in which protein concentration exceeds the solubility limit. Equilibrium is restored when aggregates or crystals are formed and the protein concentration reaches the solubility limit. Through a series of elegantly designed experiments, Goto and colleagues have demonstrated that protein aggregation is driven by the same principle of supersaturation.^36,37^ Vendruscolo and colleagues^38,39^ examined cellular protein concentrations relative to their solubility limit and found that neurodegeneration-related pathways are enriched in proteins at supersaturated concentrations.

**Fig. 1:**
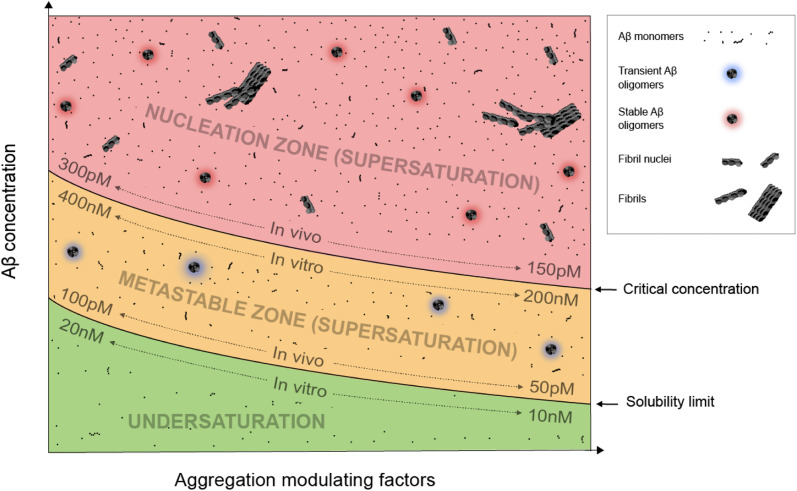
A phase diagram of Aβ supersaturation. Both Aβ concentration and environmental factors affect the phase diagram. In the undersaturation zone, Aβ exists mostly as monomers. The area of supersaturation consists of a metastable zone and a nucleation zone. In the metastable zone, Aβ exists as monomers and transient oligomers, and does not spontaneously aggregate but can aggregate in the presence of aggregate seeds. In the nucleation zone, Aβ can aggregate spontaneously and exists as a mixture of soluble Aβ monomers, stable oligomers, fibril nuclei, and fibrils.

Fig. 1 depicts an Aβ phase diagram in the context of aggregation. The Aβ solubility curve divides the phase diagram into two regions: undersaturation and supersaturation. The supersaturation region is further divided into two zones: metastable zone and nucleation zone. The boundary between the nucleation and metastable zones corresponds to the “critical concentration” for Aβ aggregation.^40,41^ Below we describe seven key points of Aβ aggregation in the framework of supersaturation.

(i) Aβ aggregation requires a supersaturated solution. Protein aggregation involves two distinct steps: fibril nucleation and growth. Fibril nucleation requires overcoming of a kinetic or energy barrier to form structurally ordered fibril nuclei and is thus the rate-limiting step. Fibril growth is an energetically favorable reaction. Both fibril nucleation and growth require supersaturation. Changes in solution pH and addition of salts, ions, or polymers are often used to alter the properties of proteins, the chemical potential of the solution, or interactions between proteins to achieve supersaturation. In a typical *in vitro* aggregation experiment, Aβ stock solutions in denaturing buffers such as urea or organic solvents such as dimethyl sulfoxide are mixed with a native buffer to immediately create a supersaturated solution. Depending on the concentration, Aβ would aggregate immediately or after a lag time.^33^

(ii) To spontaneously aggregate, Aβ concentration needs to be in the nucleation zone of supersaturation. When Aβ concentration exceeds the solubility limit, it does not immediately form the stable fibril nucleus. The energy barrier for nucleation allows Aβ concentrations to increase further from the solubility limit and into the zone of supersaturation. The supersaturation zone that results in spontaneous nucleation of Aβ fibrils is referred to as the nucleation zone. For *in vitro* aggregation, Hellstrand *et al.*^40^ reported that there was no spontaneous Aβ42 aggregation when Aβ concentration was between 10 and 200 nM. Aβ42 aggregation was observed at Aβ42 concentrations higher than 260 nM, which defines the boundary between the nucleation zone and metastable zone under their aggregation conditions.^40^

(iii) Within the nucleation zone, higher Aβ concentrations lead to faster nucleation rates. The further away from the solubility limit, the higher energy Aβ accumulates. As a result, Aβ at higher concentrations aggregate at a faster rate. Hellstrand *et al.*^40^ studied the aggregation of Aβ42 at a wide range of concentrations, and found that Aβ concentration has a linear relationship with the logarithmic value of the aggregation lag time. Aβ42 at 0.26 μM has a lag time of ∼24 h, whereas Aβ42 concentrations at >5 μM observe almost no lag time.

(iv) Aβ in the metastable zone of supersaturation does not spontaneously initiate aggregation, but can aggregate in the presence of pre-formed aggregates, often referred to as “fibril seeds”. While spontaneous fibril nucleation needs to overcome an energy barrier, fibril-seeded aggregation is a much more energetically favorable reaction. Cohen *et al.*^25^ showed that Aβ aggregation in the presence of even small amounts of amyloid fibrils is dominated by fibril-catalyzed secondary nucleation reactions, rather than the classical mechanism of primary nucleation.

(v) Once aggregation starts, it will continue until the protein concentration reaches the solubility limit. Because the supersaturation is a non-equilibrium state, initiation of protein aggregation will restore the equilibrium state of saturation, where solubilization of Aβ from fibrils and fibrillization of Aβ from monomers reach equilibrium. Hellstrand *et al.*^40^ found that, with starting concentrations ranging from 0.2 to 10 μM, the soluble Aβ concentration at the end of aggregation converge to approximately 15 nM, suggesting that Aβ42 solubility is approximately 10–20 nM for the specific aggregation conditions of their study. For *in vivo* Aβ concentrations, Portelius *et al.*^42^ found that the Aβ42 concentrations in the cerebrospinal fluid of familial Alzheimer's patients are similar to the sporadic Alzheimer's patients, even though this familial mutation has been shown to increase plasma Aβ42 levels at preclinical stage.^43^ The implications of these studies are that even though familial mutations of Alzheimer's disease changed the Aβ concentrations and thus result in increased aggregation propensity, the Aβ aggregation in the post-amyloid stage is similar to that in sporadic Alzheimer's patients because the Aβ solubility for these patients are similar.

(vi) An Aβ solution in the presence of aggregates can no longer maintain supersaturation. Due to the presence of seeded aggregation, an increase in Aβ concentration above the solubility limit will lead to aggregation. As a result, Aβ concentration can no longer maintain supersaturation. A direct *in vivo* implication of this point is that Aβ concentrations in amyloid-positive individuals cannot reach the same level as amyloid-negative individuals. After injecting isotopically-labeled Aβ into the interstitial fluid, Hong *et al.*^44^ found that the recovered Aβ from plaque-rich mice is only 45% of that from plaque-free mice, supporting the notion that most of the newly produced Aβ proteins deposit to amyloid plaques.

(vii) In the presence of a large amount of aggregates, Aβ concentration cannot become undersaturated, because the aggregates can be solubilized when protein concentration reaches below the solubility limit. For individuals that are amyloid-positive, this means that the Aβ clearance pathway will not be able to lower Aβ concentrations as much as in amyloid-negative individuals. It has been shown that, in plaque-free mice, acute inhibition of γ-secretase activity led to rapid decline of Aβ42 concentration.^44^ In contrast, plaque-rich mice showed significantly less concentration reduction, supporting the role of amyloid plaques as a reservoir of soluble Aβ.^44^

## Difference in the Aβ phase diagram for *in vitro* and *in vivo* conditions

The exact parameters that define the Aβ phase diagram under *in vitro* and *in vivo* conditions are vastly different. The extensive study by Hellstrand *et al.*^40^ of Aβ42 aggregation *in vitro* at a wide range of Aβ42 concentrations put Aβ42 solubility at approximately 10–20 nM and Aβ42 critical aggregation concentrations at approximately 200–400 nM. For *in vivo* conditions, it is not possible to perform any controlled aggregation studies. However, some parameters of the phase diagram can be implicated from biomarker studies in Alzheimer's patients. Because Aβ solubility is defined as the Aβ concentration in the presence of amyloid plaques, we used the cerebrospinal fluid (CSF) Aβ42 concentrations in the amyloid-positive individuals as an approximation of Aβ42 solubility and CSF Aβ42 concentrations in the amyloid-negative individuals as an approximation of Aβ42 critical concentrations. With these assumptions, the Aβ42 solubility *in vivo* was estimated to be 50–100 pM and Aβ critical concentration was estimated to be 150–300 pM.^15,45,46^ The Aβ42 concentration for *in vitro* aggregation differs from *in vivo* aggregation by approximately three orders of magnitude. Part of the reason for the extremely low critical concentration of *in vivo* Aβ42 aggregation may be the presence of aggregation-promoting factors such as lipids, membrane surfaces, and interacting proteins.

We note that each individual may have a distinct *in vivo* phase diagram that determines their individualized Aβ aggregation behavior. The Aβ solubility and the boundary of the nucleation zone are determined by the local concentrations of proteins, lipids, and metabolites. A wide range of Aβ concentrations have been observed in amyloid-positive individuals.^15,45^ Based on the supersaturation theory, Aβ concentrations in the presence of amyloid plaques correspond to the solubility limit, and thus these results suggest a wide range of Aβ *in vivo* solubility in different individuals.

Aβ40 modifies the phase diagram of Aβ42 aggregation by interacting with Aβ42. As a result, Aβ42/Aβ40 ratio is a more reliable descriptor of Aβ42 aggregation propensity than the absolute Aβ42 concentration alone.^47,48^ In a comprehensive study of 138 pathogenic presenilin-1 mutations, Sun *et al.*^49^ found that a quarter of the presenilin-1 variants increased production of Aβ42, and most variants producing lower levels of Aβ42 exhibited a compromised ability to produce Aβ40, leading to a higher Aβ42/Aβ40 ratio. The work of Sun *et al.*^49^ suggests that familial Alzheimer's disease mutations modulate the phase diagram of Aβ42 aggregation through not only Aβ42 concentrations, but also Aβ42/Aβ40 ratio.

## Comparison between *in vivo* and *in vitro* Aβ aggregation

**Fig. 2:**
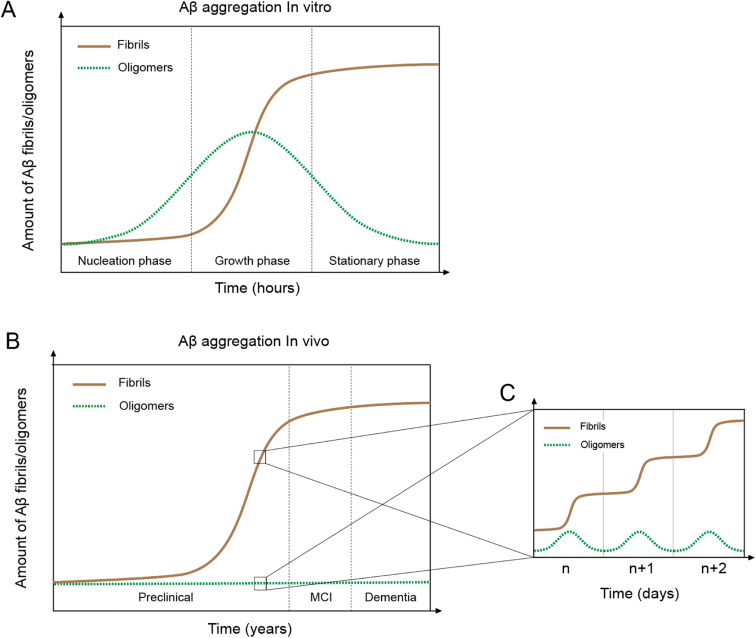
*In vivo and in vitro* Aβ aggregation curves. (A) Aβ aggregation *in vitro* leading to fibril formation (brown line) shows a typical sigmoidal curve with three phases: nucleation, growth, and stationary. Oligomers (green line) first appear in the nucleation phase but disappear towards the end of the aggregation process. (B) Aβ aggregation *in vivo* displays a similar sigmoidal curve of fibril formation as the *in vitro* system, but with fundamentally different features. (C) *In vivo* day-to-day Aβ aggregation shows a sigmoidal curve and oligomer formation.

*In vitro* Aβ aggregation kinetics are typically represented by a sigmoidal curve,^33,50^ which consists of three phases: nucleation, growth, and stationary (Fig. 2A). The rate of *in vitro* aggregation can be measured by the length of the nucleation phase, also called “lag time”. Using chemical kinetics and mathematical modeling, Dear *et al.*^51^ show that oligomers are transiently formed during the process of fibrillization and disappear towards the end of the aggregation reaction. The secondary nucleation process^50^ also leads to the formation of toxic oligomers, suggesting that oligomer formation may be an integral part of the overall Aβ aggregation process.^52^ Cryo-EM studies have revealed mechanistic insights into the fibril-catalyzed secondary nucleation.^53^

Accumulation of Aβ plaques *in vivo* appears to show a similar sigmoidal curve^21,54^ (Fig. 2B), but the nature of the *in vivo* Aβ aggregation curve is fundamentally different from the *in vitro* aggregation curve. *In vitro* Aβ aggregation is a closed system, transitioning from a non-equilibrium state consisting of a supersaturated solution to a final equilibrium state consisting of Aβ fibrils and a saturated Aβ solution. *In vivo* Aβ aggregation, on the other hand, is an open system, constantly replenishing and removing Aβ through production and clearance pathways. Because Aβ production and clearance is under the control of the 24-hour circadian clock, Aβ aggregation *in vivo* likely also has a circadian rhythm, with a daily sigmoidal aggregation curve (Fig. 2C). Aβ oligomers, due to their association with the Aβ aggregation process, are also produced as part of the daily aggregation process.

## *In vivo* Aβ concentration dynamics in the framework of Aβ supersaturation

**Fig. 3:**
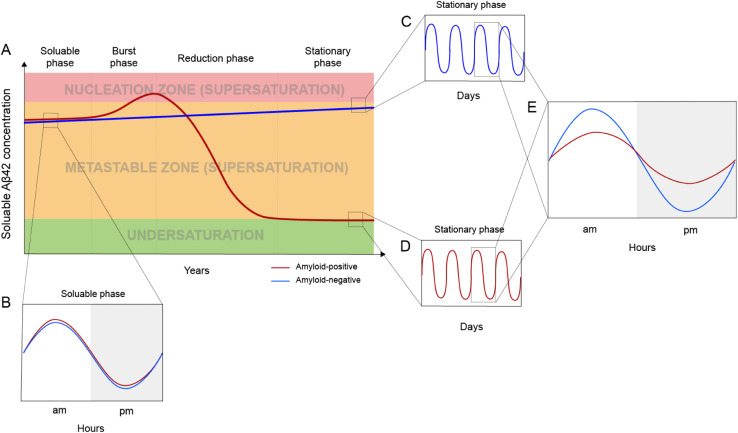
*In vivo* Aβ dynamics in the framework of supersaturation. (A) Two imaginary individuals are considered here: one eventually becomes amyloid-positive (red line) and the other one remains amyloid-negative (blue line). The Aβ concentration in the amyloid-negative individual has a slow linear increase but never goes into the nucleation zone. For the amyloid-positive individual, Aβ concentration can be divided into four phases: soluble, burst, reduction, and stationary. (B) Before amyloid formation, the amyloid-positive individual and the amyloid-negative individual display highly similar circadian fluctuations. (C and D) Daily modulation of soluble Aβ42 in amyloid-negative (C) and amyloid-positive (D) individuals. (E) After amyloid formation, the amyloid-positive individual has a dramatically reduced circadian amplitude as a result of amyloid formation.

Based on the framework of Aβ supersaturation, the Aβ concentrations of two imaginary individuals are plotted in Fig. 3A. One is an amyloid-negative individual, who never develops amyloid and dies amyloid-free. This amyloid-negative individual's Aβ concentration is simplified as a linear line, showing the overall increase in Aβ concentration over this person's adult lifetime.^55–57^ The Aβ concentration of this amyloid-negative individual stays in the metastable zone. The other imaginary person is an amyloid-positive individual who develops amyloid deposition later in life. The age-dependent changes of Aβ concentration over the amyloid-positive individual's adult lifetime can be divided into four phases: soluble phase, burst phase, reduction phase, and stationary phase (Fig. 3A). The burst phase is a prediction based on the framework of supersaturation because spontaneous Aβ aggregation requires Aβ concentration to be in the nucleation zone. This can be achieved by an accelerated increase in Aβ concentration, a “burst”, where Aβ concentration crosses the threshold into the nucleation zone (moving up along the *Y*-axis of Fig. 1). This could also be achieved by lowering the boundary of the nucleation zone because variations in *in vivo* environments can modulate the boundaries between the two zones of the supersaturation and even the solubility limit of Aβ (moving right along the *X*-axis of Fig. 1). The reduction phase is when spontaneous Aβ aggregation starts and eventually leads to a lower Aβ concentration. The stationary phase can be classified as the stage when the Aβ concentration reaches a steady-state. Mild cognitive impairment and dementia appear years or decades into the stationary phase.^58^ Studies have been performed to compare the amyloid-positive group and amyloid-negative group in the stationary phase, and show that the Aβ concentration in the amyloid-positive group is markedly lower than that in the amyloid-negative group.^15,59^ In addition to lowered Aβ concentration, amyloid formation also leads to a reduction in the amplitude of Aβ circadian fluctuations (Fig. 3E).

## Post-amyloid Aβ dynamics and circadian rhythm

Aβ concentration has a circadian rhythm.^52^ Amyloid formation leads to reduced overall Aβ42 concentration and reduced amplitude of the Aβ42 circadian rhythm. Since Aβ can no longer maintain supersaturation in the presence of plaques, the post-amyloid Aβ concentration is close to Aβ′s *in vivo* solubility. For the same individual, the post-amyloid Aβ concentration is likely lower than the pre-amyloid Aβ concentration. Bateman and colleagues^15^ studied the circadian dynamics of Aβ concentration and found that the circadian amplitude in amyloid-negative group is 15.6 pM, almost 3-fold higher than the circadian amplitude of the amyloid-positive group (6.3 pM).

Both the lowered Aβ42 concentration and diminished circadian rhythm over a long period of time may be pathogenic and contribute to cognitive decline and dementia. Aβ is an evolutionarily conserved protein,^60^ although its precise physiological function has not been conclusively established.^61^ The reduced amplitude in Aβ circadian rhythm may underlie the sleep disturbances associated with amyloid deposition.^62^ In an analysis of 598 amyloid-positive individuals, Sturchio *et al.*^63^ found that normal cognition is associated with preservation of soluble Aβ42 concentrations, suggesting that sufficient Aβ42 concentrations are critical for cognition. In a cellular model, Zhou *et al.*^64^ showed that restoring physiological amounts of Aβ in APP-deleted neurons elevated synapse number and synaptic transmission, supporting a positive role of Aβ in synapse function.

## Implications for therapeutic development

In considering the daily production and clearance of Aβ proteins *in vivo*, therapeutic treatments must be designed so as to address the constant cycle of Aβ aggregation.

### Non-linear effects of plaque removal

One important implication of the supersaturation framework is that the effect of plaque removal on Aβ aggregation and the subsequent effect on cognitive function is not linear. Due to the ability of the large aggregates to act as both fibril seeds for aggregation and reservoirs for soluble monomers, we expect that cognitive impairement would be mitigated only after a large proportion of the accumulated plaques have been cleared. Aβ aggregation in the presence of plaques *versus* in the absence of plaques is fundamentally different: one being spontaneous aggregation and the other seeded aggregation. In the framework of Aβ supersaturation, the success of the anti-amyloid therapy depends on the removal of most seeding-competent plaques so that aggregation is no longer driven by fibril seeds. By examining clinical trial data of four anti-Aβ antibodies, Karran and De Strooper^23^ reached a similar conclusion that amyloid plaque needs to be reduced to a low level to show significant clinical benefit.

### Anti-Aβ treatment

Several anti-Aβ monoclonal antibodies have advanced to late stage clinical trials or gained FDA approval as treatment options. The main mechanism of action for these antibodies is the reduction of plaque load. Surprisingly, an enormous amount of resources has been poured into developing anti-amyloid therapies, but there are no well-explained biochemical pathways that would lead from plaque reduction to cognitive improvement. Likewise, there is no clear biochemical rationalization as to how plaque reduction would lead to reduced toxic oligomer production. The supersaturation framework points to the removal of seeded Aβ aggregation as the main benefit of plaque removal, which restores Aβ42 concentration to a higher level and reduces the daily toxic assault of Aβ oligomer formation.

### Modulation of Aβ concentration

Lowering monomer Aβ concentration has long been considered as a therapeutic strategy. This can be achieved using inhibitors or modulators of β-secrease^65^ and γ-secretase.^66^ Alternatively, antibodies that bind soluble Aβ can also be used to lower Aβ levels. Recent development in this area has been reviewed in Long and Holtzman.^67^ As a standalone strategy, this approach is likely most effective in the burst phase (Fig. 3A), when an increase in Aβ concentration poses the greatest risk of initiating amyloid formation. Once amyloid is formed, the mechanism of aggregation shifts from spontaneous aggregation to seeded aggregation, and Aβ concentration plays a lesser role in the rate of aggregate formation. In the scenario where the majority of plaques and seeding-competent components have been removed, Aβ concentration will return to a supersaturated state, which can be monitored with CSF or plasma Aβ measurements.

### Personalized Aβ biomarkers

Measurements of Aβ42 in human CSF show a wide range of concentrations. Although the amyloid-positive and amyloid-negative groups can be distinguished using a cutoff of Aβ42 concentration, a large number of individuals, for example, 8% of cases in Palmqvist *et al.,*^59^ do not show agreement between Aβ42 concentration and amyloid imaging. This is likely due to large inter-individual differences in Aβ42 concentrations. One solution to this problem is to establish Aβ concentration as a personalized biomarker. Then changes in Aβ concentration can be compared to the past levels of the same individual. It has been shown that the Aβ42 concentrations in amyloid-positive and amyloid-negative cohorts differ by 2–3 fold.^15,59^ A change of this magnitude would be readily detected using the same individual's history of Aβ concentration. The personal history of Aβ concentration will be particularly useful to detect if Aβ supersaturation is restored after a therapeutic intervention that has cleared the amyloid plaques.

### Aggregation inhibitors

An additional personalized treatment strategy would be tailored to the specific pattern of aggregation exhibited by the patient. Due to the difference between spontaneous and seeded aggregation, two types of aggregation inhibitors may be needed. Spontaneous aggregation inhibitors are most important in the burst phase before a significant amount of amyloids have built up. Once the seeded aggregation becomes the dominant mechanism, inhibitors for seeded aggregation will work more effectively.

### Toxicity blockers

Proteins or small molecules that bind directly to toxic species can serve as toxicity blockers. This class of therapeutic molecules would be effective throughout the course of Alzheimer's disease. It may be particularly helpful in combination with anti-amyloid therapy, which by itself does not eliminate the toxicity of soluble Aβ. However, these types of potential drugs are also the most elusive due to a lack of understanding of both mechanisms of toxicity and the structures of the toxic Aβ species.

### Different therapeutic windows call for different treatment strategies

Due to the high degree of variations in Aβ aggregation behavior at different stages of pathogenesis, therapeutic strategies will need to be adjusted accordingly. In the soluble and burst phase, the most effective way to reduce the risk of Aβ aggregation is to keep Aβ levels away from the nucleation zone. This can be done either by reduction of soluble Aβ concentration (*e.g.*, β- and γ-secretase inhibitors, Aβ immunization) or modulate the Aβ phase diagram by increasing the boundary concentration between metastable and nucleation zones. In the reduction phase, Aβ aggregation has started and the presence of small amounts of amyloid plaques provides the best opportunity for anti-amyloid therapy. Reduction of soluble Aβ concentration is likely not effective in the reduction phase because Aβ aggregation is driven by fibril-catalyzed secondary nucleation. Because oligomer formation is associated with the overall aggregation process, toxicity blockers will also be desired to limit damage to synaptic connections and neuronal cells. The stationary phase is the least desired treatment window because the effect of treatment will only be felt after the vast majority of plaques have been removed.

## Issues of interest for future investigations

In order to further our understanding of Alzheimer's disease and other amyloid-related disorders, in the light of the supersaturation framework, future investigation could expand on the following ideas. First, there is a wide range of Aβ42 concentrations in amyloid-negative individuals. It is important to distinguish whether a higher Aβ concentration means a higher risk of imminent aggregation or if it indicates that the individual has a higher tolerance to Aβ aggregation, in other words, a higher boundary for the nucleation zone. It is conceivable that different individuals have their unique combination of aggregation-promoting and inhibiting factors and some may be more tolerant to higher Aβ concentrations than others. Identifying these aggregation-inhibiting factors may provide a new form of therapeutic intervention. Second, Aβ40 has been shown to be an important and likely the best-characterized inhibitor of *in vivo* Aβ42 aggregation. Mutations in familial Alzheimer's disease often lead to an increase in Aβ42/Aβ40 ratio, not simply increased Aβ42 concentrations. In sporadic Alzheimer's disease, Aβ42/Aβ40 ratio is a better predictor of Alzheimer's risk than the absolute Aβ42 concentration. It is likely that this modulation of the Aβ42 phase diagram is a result of a direct interaction with Aβ40. Therefore, exploring the potential use of Aβ40 or another Aβ variant as a modulator of Aβ42 aggregation deserves further investigation. Third, as a consequence of Aβ42 aggregation, the net concentration of Aβ42 is lowered as it is no longer able to maintain supersaturation. Although the exact physiological function of Aβ42 is not clear, the reduction in both absolute Aβ42 concentration and its circadian amplitude may have a negative effect on cognition, especially over a long period of time. While replenishment of Aβ42 is out of the question due to seeded aggregation, identification of a functionally equivalent and non-aggregating form of Aβ42 may provide another disease-modifying treatment.

## Author contributions

ZG: conceptualization, analysis, funding acquisition, investigation, visualization, writing of original draft, manuscript editing and revision; DPB: analysis, investigation, visualization, manuscript editing and revision.

## Conflicts of interest

Nothing declared.

## Supplementary Material

## References

[cit1] Buxbaum J. N., Dispenzieri A., Eisenberg D. S., Fändrich M., Merlini G., Saraiva M. J. M., Sekijima Y., Westermark P. (2022). Amyloid.

[cit2] Sinnige T., Stroobants K., Dobson C. M., Vendruscolo M. (2020). Q. Rev. Biophys..

[cit3] Chiti F., Dobson C. M. (2017). Annu. Rev. Biochem..

[cit4] Xue C., Lin T. Y., Chang D., Guo Z. (2017). R. Soc. Open Sci..

[cit5] Groenning M. (2010). J. Chem. Biol..

[cit6] Gallardo R., Ranson N. A., Radford S. E. (2020). Curr. Opin. Struct. Biol..

[cit7] Sawaya M. R., Hughes M. P., Rodriguez J. A., Riek R., Eisenberg D. S. (2021). Cell.

[cit8] Fitzpatrick A. W., Saibil H. R. (2019). Curr. Opin. Struct. Biol..

[cit9] Selkoe D. J. (2011). Cold Spring Harb. Perspect. Biol..

[cit10] Golde T. E. (2022). Mol. Neurodegener..

[cit11] Congdon E. E., Sigurdsson E. M. (2018). Nat. Rev. Neurol..

[cit12] Knopman D. S., Amieva H., Petersen R. C., Chételat G., Holtzman D. M., Hyman B. T., Nixon R. A., Jones D. T. (2021). Nat. Rev. Dis. Primers.

[cit13] De Strooper B., Vassar R., Golde T. (2010). Nat. Rev. Neurol..

[cit14] Mehta P. D., Pirttilä T., Mehta S. P., Sersen E. A., Aisen P. S., Wisniewski H. M. (2000). Arch. Neurol..

[cit15] Huang Y., Potter R., Sigurdson W., Santacruz A., Shih S., Ju Y.-E., Kasten T., Morris J. C., Mintun M., Duntley S., Bateman R. J. (2012). Arch. Neurol..

[cit16] Iwatsubo T., Odaka A., Suzuki N., Mizusawa H., Nukina N., Ihara Y. (1994). Neuron.

[cit17] Miller D. L., Papayannopoulos I. A., Styles J., Bobin S. A., Lin Y. Y., Biemann K., Iqbal K. (1993). Arch. Biochem. Biophys..

[cit18] Hardy J. A., Higgins G. A. (1992). Science.

[cit19] Hardy J., Selkoe D. J. (2002). Science.

[cit20] Hardy J. (2006). J. Alzheimers Dis..

[cit21] Selkoe D. J., Hardy J. (2016). EMBO Mol. Med..

[cit22] Karran E., Mercken M., Strooper B. D. (2011). Nat. Rev. Drug Discov..

[cit23] Karran E., De Strooper B. (2022). Nat. Rev. Drug Discov..

[cit24] Cline E. N., Bicca M. A., Viola K. L., Klein W. L. (2018). J. Alzheimers Dis..

[cit25] Cohen S. I. A., Linse S., Luheshi L. M., Hellstrand E., White D. A., Rajah L., Otzen D. E., Vendruscolo M., Dobson C. M., Knowles T. P. J. (2013). Proc. Natl. Acad. Sci. U.S.A..

[cit26] Mahase E. (2023). BMJ.

[cit27] Knopman D. S., Jones D. T., Greicius M. D. (2021). Alzheimer’s Dementa.

[cit28] Sabbagh M. N., Cummings J. (2021). Alzheimer’s Dementa.

[cit29] van Dyck C. H., Swanson C. J., Aisen P., Bateman R. J., Chen C., Gee M., Kanekiyo M., Li D., Reyderman L., Cohen S., Froelich L., Katayama S., Sabbagh M., Vellas B., Watson D., Dhadda S., Irizarry M., Kramer L. D., Iwatsubo T. (2023). N. Engl. J. Med..

[cit30] Hardy J., Mummery C. (2023). Brain.

[cit31] Weglinski C., Jeans A. (2023). Neuronal Signaling.

[cit32] Sims J. R., Zimmer J. A., Evans C. D., Lu M., Ardayfio P., Sparks J., Wessels A. M., Shcherbinin S., Wang H., Monkul Nery E. S., Collins E. C., Solomon P., Salloway S., Apostolova L. G., Hansson O., Ritchie C., Brooks D. A., Mintun M., Skovronsky D. M., TRAILBLAZER-ALZ 2 Investigators (2023). JAMA.

[cit33] Harper J. D., Lansbury P. T. (1997). Annu. Rev. Biochem..

[cit34] Coquerel G. (2014). Chem. Soc. Rev..

[cit35] McPherson A., Gavira J. A. (2013). Acta Crystallogr., Sect. F Struct. Biol. Commun..

[cit36] So M., Hall D., Goto Y. (2016). Curr. Opin. Struct. Biol..

[cit37] Goto Y., Noji M., Nakajima K., Yamaguchi K. (2022). Molecules.

[cit38] Ciryam P., Tartaglia G. G., Morimoto R. I., Dobson C. M., Vendruscolo M. (2013). Cell Rep..

[cit39] Ciryam P., Kundra R., Morimoto R. I., Dobson C. M., Vendruscolo M. (2015). Trends Pharmacol. Sci..

[cit40] Hellstrand E., Boland B., Walsh D. M., Linse S. (2010). ACS Chem. Neurosci..

[cit41] Novo M., Freire S., Al-Soufi W. (2018). Sci. Rep..

[cit42] Portelius E., Andreasson U., Ringman J. M., Buerger K., Daborg J., Buchhave P., Hansson O., Harmsen A., Gustavsson M. K., Hanse E., Galasko D., Hampel H., Blennow K., Zetterberg H. (2010). Mol. Neurodegener..

[cit43] Ringman J. M., Younkin S. G., Pratico D., Seltzer W., Cole G. M., Geschwind D. H., Rodriguez-Agudelo Y., Schaffer B., Fein J., Sokolow S., Rosario E. R., Gylys K. H., Varpetian A., Medina L. D., Cummings J. L. (2008). Neurology.

[cit44] Hong S., Quintero-Monzon O., Ostaszewski B. L., Podlisny D. R., Cavanaugh W. T., Yang T., Holtzman D. M., Cirrito J. R., Selkoe D. J. (2011). J. Neurosci..

[cit45] Hansson O., Zetterberg H., Buchhave P., Londos E., Blennow K., Minthon L. (2006). Lancet Neurol..

[cit46] Oe T., Ackermann B. L., Inoue K., Berna M. J., Garner C. O., Gelfanova V., Dean R. A., Siemers E. R., Holtzman D. M., Farlow M. R., Blair I. A. (2006). Rapid Commun. Mass Spectrom..

[cit47] Doecke J. D., Pérez-Grijalba V., Fandos N., Fowler C., Villemagne V. L., Masters C. L., Pesini P., Sarasa M., AIBL Research Group (2020). Neurology.

[cit48] Delaby C., Estellés T., Zhu N., Arranz J., Barroeta I., Carmona-Iragui M., Illán-Gala I., Santos-Santos M. Á., Altuna M., Sala I., Sánchez-Saudinós M. B., Videla L., Valldeneu S., Subirana A., Tondo M., Blanco-Vaca F., Lehmann S., Belbin O., Blesa R., Fortea J., Lleó A., Alcolea D. (2022). Alzheimers Res. Ther..

[cit49] Sun L., Zhou R., Yang G., Shi Y. (2017). Proc. Natl. Acad. Sci. U. S. A..

[cit50] Törnquist M., Michaels T. C. T., Sanagavarapu K., Yang X., Meisl G., Cohen S. I. A., Knowles T. P. J., Linse S. (2018). Chem. Commun..

[cit51] Dear A. J., Michaels T. C. T., Meisl G., Klenerman D., Wu S., Perrett S., Linse S., Dobson C. M., Knowles T. P. J. (2020). Proc. Natl. Acad. Sci. U. S. A..

[cit52] Cohen S. I. A., Arosio P., Presto J., Kurudenkandy F. R., Biverstål H., Dolfe L., Dunning C., Yang X., Frohm B., Vendruscolo M., Johansson J., Dobson C. M., Fisahn A., Knowles T. P. J., Linse S. (2015). Nat. Struct. Mol. Biol..

[cit53] Törnquist M., Cukalevski R., Weininger U., Meisl G., Knowles T. P. J., Leiding T., Malmendal A., Akke M., Linse S. (2020). Proc. Natl. Acad. Sci. U.S.A..

[cit54] Jack Jr C. R., Knopman D. S., Jagust W. J., Shaw L. M., Aisen P. S., Weiner M. W., Petersen R. C., Trojanowski J. Q. (2010). Lancet Neurol..

[cit55] Mengel D., Liu W., Glynn R. J., Selkoe D. J., Strydom A., Lai F., Rosas H. D., Torres A., Patsiogiannis V., Skotko B., Walsh D. M. (2020). Alzheimers Res. Ther..

[cit56] Zecca C., Pasculli G., Tortelli R., Dell'Abate M. T., Capozzo R., Barulli M. R., Barone R., Accogli M., Arima S., Pollice A., Brescia V., Logroscino G. (2021). Front. Aging Neurosci..

[cit57] de Wolf F., Ghanbari M., Licher S., McRae-McKee K., Gras L., Weverling G. J., Wermeling P., Sedaghat S., Ikram M. K., Waziry R., Koudstaal W., Klap J., Kostense S., Hofman A., Anderson R., Goudsmit J., Ikram M. A. (2020). Brain.

[cit58] Hadjichrysanthou C., Evans S., Bajaj S., Siakallis L. C., McRae-McKee K., de Wolf F., Anderson R. M., Alzheimer's Disease Neuroimaging Initiative (2020). Alzheimers Res. Ther..

[cit59] Palmqvist S., Zetterberg H., Blennow K., Vestberg S., Andreasson U., Brooks D. J., Owenius R., Hägerström D., Wollmer P., Minthon L., Hansson O. (2014). JAMA Neurol..

[cit60] Tharp W. G., Sarkar I. N. (2013). BMC Genomics.

[cit61] Kent S. A., Spires-Jones T. L., Durrant C. S. (2020). Acta Neuropathol..

[cit62] Wang C., Holtzman D. M. (2020). Neuropsychopharmacology.

[cit63] Sturchio A., Dwivedi A. K., Young C. B., Malm T., Marsili L., Sharma J. S., Mahajan A., Hill E. J., Andaloussi S. E., Poston K. L., Manfredsson F. P., Schneider L. S., Ezzat K., Espay A. J. (2021). eClinicalMedicine.

[cit64] Zhou B., Lu J. G., Siddu A., Wernig M., Südhof T. C. (2022). Sci. Transl. Med..

[cit65] Ghosh A. K., Osswald H. L. (2014). Chem. Soc. Rev..

[cit66] Golde T. E., Koo E. H., Felsenstein K. M., Osborne B. A., Miele L. (2013). Biochim. Biophys. Acta.

[cit67] Long J. M., Holtzman D. M. (2019). Cell.

